# Design, Motivation and Evaluation of a Full-Resolution Optical Tactile Sensor

**DOI:** 10.3390/s19040928

**Published:** 2019-02-22

**Authors:** Carmelo Sferrazza, Raffaello D’Andrea

**Affiliations:** Institute for Dynamic Systems and Control, ETH Zurich, 8092 Zurich, Switzerland; rdandrea@ethz.ch

**Keywords:** tactile sensing, machine learning, optical tactile sensors, computer vision, robotics

## Abstract

Human skin is capable of sensing various types of forces with high resolution and accuracy. The development of an artificial sense of touch needs to address these properties, while retaining scalability to large surfaces with arbitrary shapes. The vision-based tactile sensor proposed in this article exploits the extremely high resolution of modern image sensors to reconstruct the normal force distribution applied to a soft material, whose deformation is observed on the camera images. By embedding a random pattern within the material, the full resolution of the camera can be exploited. The design and the motivation of the proposed approach are discussed with respect to a simplified elasticity model. An artificial deep neural network is trained on experimental data to perform the tactile sensing task with high accuracy for a specific indenter, and with a spatial resolution and a sensing range comparable to the human fingertip.

## 1. Introduction

Relatively small image sensors nowadays provide very high resolutions and wide dynamic ranges. Together with cost effectiveness, these factors have made cameras a comprehensive solution for providing robots with a sense of vision. Moreover, in particular in recent years, the use of machine learning for computer vision problems has contributed to the accomplishment of numerous challenging tasks in the field (see, for example, [1,2,3]).

The benefits of artificial vision systems, from the points of view of both hardware and developed algorithms, can be exploited in different domains, as is the case for robotic tactile sensing systems. Although the fundamental importance of the sense of touch for interacting with the environment has been shown in both humans (see [4]), and robots (see [5]), finding a sensing solution that yields satisfactory performance for various types of interactions and tasks is still an open problem. By using a camera to monitor various physical properties, such as the change in light intensity, which are related to the deformation of a soft material subject to external forces, it is possible to design algorithms which reconstruct the force distribution with high resolution.

**Figure 1:**
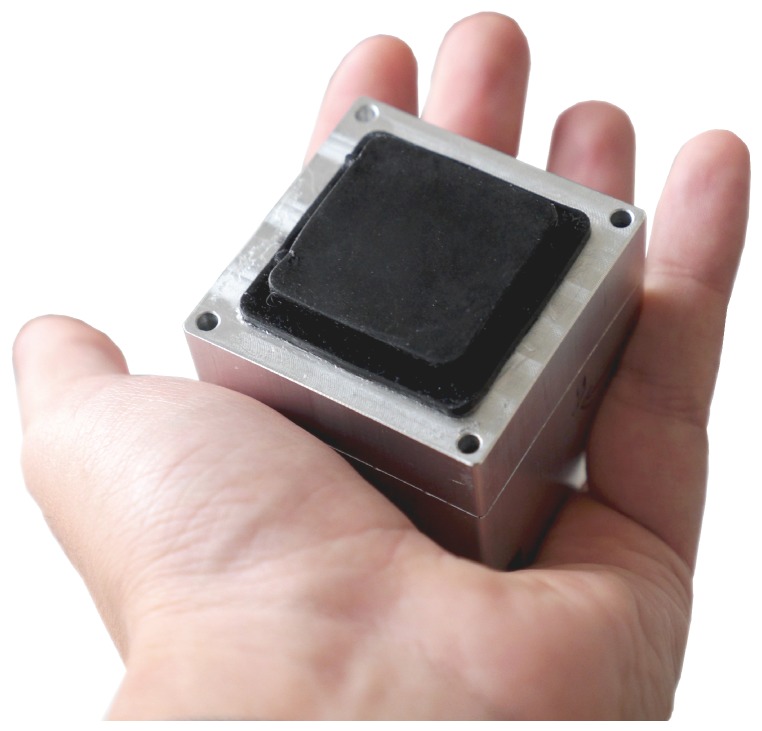
The tactile sensor presented in this article.

This article describes the design of a sensor (shown in Figure 1) that consists of a camera that tracks the movement of spherical markers within a gel, providing an approximation of the strain field inside the material. This information is exploited to reconstruct the normal external force distribution that acts on the surface of the gel. The use of a camera intrinsically renders a very high spatial resolution, given by the extensive number of pixels composing the images. A specific choice of relevant features leverages the full resolution of the camera, with the sensor’s spatial resolution not limited by the number of markers.

Moreover, this article provides a theoretical analysis of the elastic model of the material, which evaluates different marker layouts. This analysis indicates that the presence of markers at different depths within the gel yields a higher robustness to errors in the marker tracking, while retaining a small sensor threshold.

The map between the marker displacements and the normal force distribution is modeled with a neural network, which is trained on a vast number of images. These images are collected while an automatically controlled milling machine presses an indenter’s tip against the sensor’s surface at different locations. During this procedure, ground truth force measurements are provided by a force torque (F/T) sensor. The proposed approach is also discussed with respect to transfer learning applications in the author’s work in [6], where preliminary results show that it is possible to greatly reduce the required training data and the training times.

The resulting pipeline runs in real-time on a standard laptop (dual-core, 2.80 GHz), predicting the normal force distribution from the image stream at 60 Hz.

### 1.1. Related Work

As highlighted in [7], among the reasons that have delayed the widespread deployment of tactile sensors on robotic systems compared to vision sensors, the lack of a tactile analog to optical arrays is a result of the inherent complexity of interpreting the information obtained via physical contact.

In the literature, different categories of tactile sensors focus on obtaining this information using different principles (see, for example, [8], where the electrical resistance of an elastomer is related to the pressure exerted on it, and [9], where an array of zinc oxide nanorods generates a voltage signal whose amplitude is proportional to the normal force applied). A detailed description of the main classes of tactile sensors for robotic applications is provided in [10].

Optical (or vision-based) tactile sensors are a large class of devices that exploit various light-related principles, which describe how different properties change with the stress applied to a contact surface. Several examples are described in the literature, based on principles such as photometric stereo [11], total internal reflection [12], and reflected light intensity [13]. Vision-based tactile sensors only marginally affect the observed sensor’s surface, and therefore do not alter the softness of the contact area. This is a main requirement for tactile sensors that interact with the environment [7]. In fact, a soft material has the advantage of compliance, friction and conformation to the surfaces it interacts with, which for example are crucial properties for manipulation tasks.

Several approaches proposed for optical tactile sensing are based on tracking a series of markers on the sensor’s surface (see [14]). The movement of these markers is directly related to the strain field of the material, and can therefore be used to reconstruct the external force distribution on the surface. For example, in [15], a numerical method which captures a linear elastic model of the material is used to reconstruct the force distribution from marker displacements.

The choice of the marker layout in vision-based tactile sensors is investigated in various works. A symmetrical pattern is exploited in [16] to generalize tactile stimuli to new orientations. In [17], an analytical model approximately reconstructs the force distribution from the displacement of spherical markers. These markers are placed over two different depth layers within the sensor’s surface, and this layout shows a better robustness to noise compared to the case in which all markers are placed at the same depth.

To overcome the limitations imposed by the assumptions made on the material model, whose full nonlinear description is highly complex to derive, machine learning approaches have been explored for tactile sensing. By training a learning algorithm with an extensive amount of data, it is possible to reconstruct the force applied to the sensor’s surface. For example, in [18], a deep neural network is used to estimate the total contact force that an optical tactile sensor with markers printed on the surface is exerting on several objects. The need for training data is fulfilled in different ways in the literature, for instance with robot manipulators [5] or with other automatic machines [19].

This article presents an optical tactile sensor that is based on tracking spherical markers, which are randomly spread over the entire three-dimensional volume of its soft surface. The sensor presented here does not rely on tracking a specific marker layout (as in [16,17]) and is provided with standard lighting conditions (opposed to [18]), facilitating the deployment to larger surfaces (through multiple cameras) of arbitrary shapes. In fact, the proposed strategy only requires the presence of distinct features, which move according to the material deformation, therefore greatly simplifying manufacture.

Conversely to most of the cited approaches, the features engineered for the proposed supervised learning algorithm can be chosen in a way that the information at all pixels is processed, independent of the pattern choice, therefore actually exploiting the full resolution of the camera. Moreover, the proposed design and strategy can be easily extended to different types of forces and interactions.

### 1.2. Outline

The tactile sensor is presented in Section 2, where its design and the production procedure are discussed. Theoretical analyses of the sensor threshold and robustness are explained in Section 3. Section 4 describes a procedure for training data collection and the related data processing for the generation of ground truth labels. Two feature engineering strategies, both suitable for real-time execution, are presented in Section 5. The learning architecture, which captures a map between the engineered features and the generated ground truth labels, is discussed in Section 6. Results and comparisons are shown in Section 7. Finally, Section 8 draws the conclusions of the article.

## 2. Design and Production

**Figure 2:**
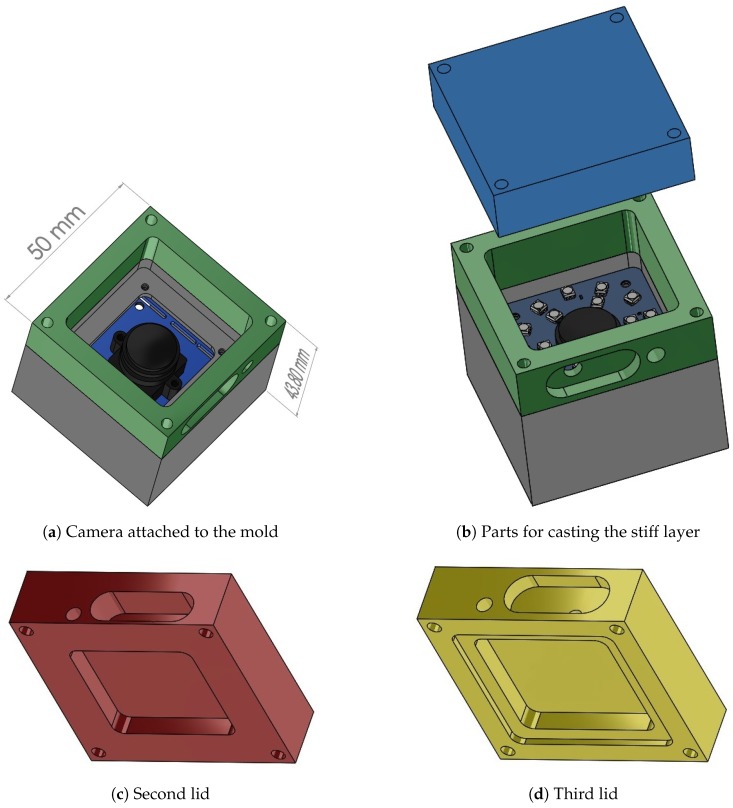
CAD drawings showing the different parts of the mold: (**a**) the camera was attached to the mold, and (**b**) the LED board was placed around the lens. Three lids (blue (**b**); red (**c**); and yellow (**d**)) were interchanged for the various production steps. The colors in this figure are only for ease of visualization, and do not correspond to the colors of the real tactile sensor.

To track the spherical markers within the soft material, a fisheye RGB camera (2.24 megapixels ELP USBFHD06H) was placed inside a mold, as shown in Figure 2a, and equipped with a board that controls two rings of LEDs (see Figure 2b), which provide constant illumination. The camera can read image frames up to 100 fps at a resolution of 640 × 480 pixels.

The soft materials employed in the sensor production were all degassed in a vacuum (to remove air bubbles, which form during mixing) and poured through cavities in the mold. The mold was placed on one of its sides (that is, with the camera lens pointing sideways) during production. Three different lids were used to perform the three respective steps of the production procedure, which is explained in the following. The design proposed here differs from the one employed in [6], where a bottom-up procedure yielded imperfections on the sensor’s top surface.

**Figure 3:**
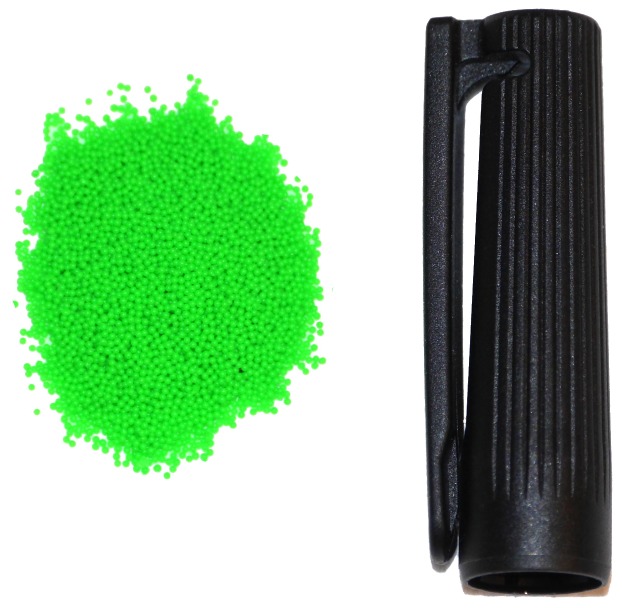
A concentration of the spherical fluorescent green markers, compared to the size of a regular pen cap.

**Figure 4:**
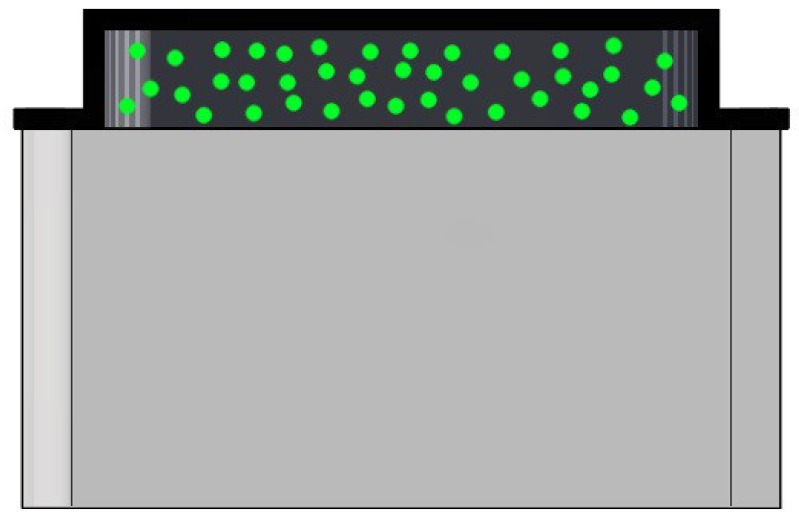
Schematic cross-sectional view of the resulting soft materials, shown here on top of each other. For ease of visualization, the base stiff layer is shown in semi-transparent light gray. The green particles were embedded in a soft layer, which was covered by an additional black layer.

A first lid closes the mold, and a relatively stiff transparent silicone layer (ELASTOSIL^®^ RT 601 RTV-2, mixing ratio 7:1, shore hardness 45A) was then poured into the mold through a side cavity, which is shown in Figure 2b. After the silicone cured, the lid was removed and replaced with the second one, shown (in red) in Figure 2c, which had an indent of 30 × 30 × 4.5 mm. Spherical fluorescent green particles (shown in Figure 3) with a diameter of 500–600 μm were mixed with a very soft silicone gel (Ecoflex™ GEL, mixing ratio 1:1, shore hardness 000-35) and poured into the mold filling the empty indent. After this soft layer was also cured, the second lid was replaced with the third one, shown (in yellow) in Figure 2d, with an indent that left an empty section around the gel of varying thickness (depending on the section) between 1 mm and 1.5 mm. A black silicone layer (ELASTOSIL^®^ RT 601 RTV-2, mixing ratio 25:1, shore hardness 10A) was then poured through the cavity on the third lid. Figure 4 shows a schematic cross-sectional view of the three silicone layers, and an example of the resulting tactile sensor is shown in Figure 1.

The stiff layer, which was poured first, served as a base for the softer materials that were placed on top of it, and as a spacer between the camera and the region of interest. All the materials employed have comparable refraction indexes, therefore preventing unwanted reflections from the LEDs. The spherical particles have a density that is close to the density of the gel they are mixed with. Together with the viscosity of the material, this is crucial to obtain a homogeneous spread of the markers over the entire depth of the gel. A detailed discussion on this point can be found in Appendix A. The black silicone layer added consistency to the extremely soft gel, which also tended to stick on contact, and provided a shield against external light disturbances. The thickness of the sensor from the base of the camera to the top of the surface is 37 mm.

## 3. Motivation

Besides ease of manufacture and portability to surfaces with arbitrary shapes, the approach presented here showed theoretical benefits in a simplified scenario. This section presents first order analyses of two different properties of the proposed sensor: the robustness to noise in the marker displacements and the sensor threshold. Here, the threshold is defined as the minimum force that leads to a detectable change in the camera image. To this purpose, simplified models of the camera and the material were considered. Since the resulting expressions were dependent on the marker distribution and on the displacements observed, Monte Carlo simulations (i.e., based on repeated random sampling) were performed for different external forces and marker layouts, and the results are discussed. In particular, four layout classes were considered:

**Single** **layer** **at** **1** **mm:** The markers were randomly distributed on a single layer (with an horizontal section of 30 mm × 30 mm) placed at a depth of 1 mm from the sensor’s surface.

**Single** **layer** **at** **2** **mm:** The markers were randomly distributed on a single layer (with an horizontal section of 30 mm × 30 mm) placed at a depth of 2 mm from the sensor’s surface.

**Single** **layer** **at** **6** **mm:** The markers were randomly distributed on a single layer (with an horizontal section of 30 mm × 30 mm) placed at a depth of 6 mm from the sensor’s surface.

**Homogeneous** **spread** **between** **1** **mm** **and** **6** **mm:** The markers were randomly distributed over a depth range of 1–6 mm from the sensor’s surface (covering an horizontal section of 30 mm × 30 mm).

In the reminder of this article, vectors are expressed as tuples for ease of notation, with dimension and stacking clear from the context.

### 3.1. Model

A semi-infinite linear elastic half-space model was assumed for the surface material. This model has shown limited loss of accuracy in the force reconstruction task for indentations in the proximity of the origin of the half-space (see [20]), and provides a tractable analytical solution. Although a simple scenario with a single rubber layer was considered, the physical parameters of the conducted simulations were chosen to approximate the first-order behavior of the sensor presented in Section 2.

Three Cartesian axes, *x*, *y* and *z*, defined the world coordinate frame. These were centered at the origin of the half-space, placed on the top surface. The *z*-axis was positive in the direction entering the material, and the remaining two axes spanned the horizontal surface. Let $s_{j}:=\left(x_{j},y_{j},z_{j}\right)$ be the position of a marker *j* before the force is applied, for $j=0,\cdots ,N_{m}-1$, where $N_{m}$ is the number of markers within the gel. Given a point force applied at the origin (this model can be easily extended to the entire force distribution, as shown in [17]), the three-dimensional displacement $\Delta s_{j}$ of the marker *j* can be derived from the Bousinnesq and Cerruti solutions (see [21]), as,

(1) $$\begin{array}{c} \Delta s_{j}=H_{j}F, \end{array}$$

where $F\in R^{3}$ is the applied force vector, and,

(2) $$H_{j}=\frac{1+\nu }{2\pi ER_{j}}[\begin{array}{ccc} \frac{R_{j}^{2}+x_{j}^{2}}{R_{j}^{2}}+\left(1-2\nu \right)\frac{R_{j}^{2}+R_{j}z_{j}-x_{j}^{2}}{{\left(R_{j}+z_{j}\right)}^{2}} & \frac{x_{j}y_{j}}{R_{j}^{2}}-\left(1-2\nu \right)\frac{x_{j}y_{j}}{{\left(R_{j}+z_{j}\right)}^{2}} & \frac{x_{j}z_{j}}{R_{j}^{2}}-\left(1-2\nu \right)\frac{x_{j}}{R_{j}+z_{j}}\\ \frac{x_{j}y_{j}}{R_{j}^{2}}-\left(1-2\nu \right)\frac{x_{j}y_{j}}{{\left(R_{j}+z_{j}\right)}^{2}} & \frac{R_{j}^{2}+y_{j}^{2}}{R_{j}^{2}}+\left(1-2\nu \right)\frac{R_{j}^{2}+R_{j}z_{j}-y_{j}^{2}}{{\left(R_{j}+z_{j}\right)}^{2}} & \frac{y_{j}z_{j}}{R_{j}^{2}}-\left(1-2\nu \right)\frac{y_{j}}{R_{j}+z_{j}}\\ \frac{x_{j}z_{j}}{R_{j}^{2}}+\left(1-2\nu \right)\frac{x_{j}}{R_{j}+z_{j}} & \frac{y_{j}z_{j}}{R_{j}^{2}}+\left(1-2\nu \right)\frac{y_{j}}{R_{j}+z_{j}} & \frac{z_{j}^{2}}{R_{j}^{2}}+2\left(1-\nu \right) \end{array}],$$

where ν and *E* are the Poisson’s ratio and the Young’s modulus of the material, respectively, and $R_{j}:=\sqrt{x_{j}^{2}+y_{j}^{2}+z_{j}^{2}}$.

A pinhole camera model (see [22] (p. 49)) was assumed, with square pixels and the optical center projected at the origin of the image coordinate frame, on the *z*-axis of the world frame. The image plane was parallel to the sensor’s surface.

The displacement $\Delta p_{j}\in R^{2}$, as observed by the camera and expressed in pixels in the image frame, was computed as the difference between the marker positions in the image after and before the force is applied. This leads to,

(3) $$\begin{array}{cc} \Delta p_{j} & =K_{a,j}\left(s_{j}+\Delta s_{j}\right)-K_{b,j}s_{j}\\ & =\left(K_{a,j}-K_{b,j}\right)s_{j}+K_{a,j}H_{j}F, \end{array}$$

where

(4) $$\begin{array}{c} K_{a,j}=\left[\begin{array}{ccc} \frac{f}{d-(z_{j}+H_{j,3}F)} & 0 & 0\\ 0 & \frac{f}{d-(z_{j}+H_{j,3}F)} & 0 \end{array}\right], \end{array}$$

(5) $$\begin{array}{c} K_{b,j}=\left[\begin{array}{ccc} \frac{f}{d-z_{j}} & 0 & 0\\ 0 & \frac{f}{d-z_{j}} & 0 \end{array}\right], \end{array}$$

*f* is the focal length of the camera (in pixels), *d* is the distance of the optical center from the origin of the world coordinate frame, and $H_{j,i}$ represents the *i*th row of the $H_{j}$ matrix, for i=1,2,3. Note that, for small *d*, which is usually the case for state-of-the-art optical tactile sensors, the difference between $K_{a,j}$ and $K_{b,j}$ is not negligible. The expression in Equation (3) can be rearranged, as explained in Appendix B, as,

(6) $$\begin{array}{cc} \Delta p_{j}= & \underset{:=P_{j}\left(\Delta p_{j}\right)}{\underset{︸}{\left\{\frac{\Delta p_{j}}{d-z_{j}}H_{j,3}+\frac{f}{{\left(d-z_{j}\right)}^{2}}\left[\begin{array}{c} x_{j}\\ y_{j} \end{array}\right]H_{j,3}+\frac{f}{d-z_{j}}\left[\begin{array}{c} H_{j,1}\\ H_{j,2} \end{array}\right]\right\}}}F=P_{j}\left(\Delta p_{j}\right)\cdot F, \end{array}$$

where $P_{j}\in R^{2\times 3}$ depends on the displacement $\Delta p_{j}$. For force sensing applications, once the marker displacement $\Delta p_{j}$ is observed in the image, the equality in Equation (6) represents an underdetermined system of two equations with three unknowns (the components of *F*). To find a unique solution, and to improve the reconstruction error in the presence of noise, more equations can be provided by tracking the remaining markers, yielding,

(7) $$\begin{array}{c} \Delta p:=\left[\begin{array}{c} \Delta p_{0}\\ \vdots \\ \Delta p_{N_{m}-1} \end{array}\right]=\underset{:=P(\Delta p)}{\underset{︸}{\left[\begin{array}{c} P_{0}\left(\Delta p_{0}\right)\\ \vdots \\ P_{N_{m}-1}\left(\Delta p_{N_{m}-1}\right) \end{array}\right]}}\cdot F=P\left(\Delta p\right)\cdot F, \end{array}$$

with $P\in R^{2N_{m}\times 3}$. The force *F* can then be reconstructed as,

(8) $$\begin{array}{c} F=P{\left(\Delta p\right)}^{†}\cdot \Delta p, \end{array}$$

where the apex ${}^{†}$ indicates the Moore–Penrose pseudo-inverse matrix. The matrix *P* depends on the different $\Delta p_{j}$, therefore the equality in Equation (8) indicates a nonlinear dependence between *F* and Δp.

The parameters used for the Monte Carlo simulations described in the following subsections are summarized in Table 1. In particular, the Young’s modulus was chosen as the linear combination of the resulting values for the soft layers described in Section 2, according to the conversions introduced in [23] (note, however, that this value is only a multiplicative factor in Equation (2), and does not have a large effect on the resulting trends in the simulations).

### 3.2. Robustness to Noise

To a certain extent, the learning algorithm presented in Section 6 aims to approximate a map similar to the one in Equation (8), which relates the observed marker displacements to the applied external force. For the purpose of analyzing how the output of this map reacts to a perturbation δp of the displacements Δp, a first order linearization was performed around the unperturbed state. Therefore, the force corresponding to the perturbed displacements can be expressed as,

(9) $$\begin{array}{c} F{|}_{\Delta p+\delta p}\approx P{\left(\Delta p\right)}^{†}\cdot \Delta p+J\left(\Delta p\right)\cdot \delta p, \end{array}$$

where *J* is the appropriate Jacobian matrix, containing the derivative terms of the map with respect to Δp. Using the properties of the norm,

(10) $$\begin{array}{c} {∥F{|}_{\Delta p+\delta p}-F{|}_{\Delta p}∥}_{2}\approx {∥J\left(\Delta p\right)\delta p∥}_{2}\leq \underset{:=\kappa (\Delta p)}{\underset{︸}{{∥J\left(\Delta p\right)∥}_{F}}}{∥\delta p∥}_{2}, \end{array}$$

where the $L^{2}$-norm and the Frobenius norm are used accordingly. The value that κ∈R takes is often referred to as the *absolute condition number* of a function (see [24] (p. 221)), and it provides a bound on the slope at which a function changes given a change in the input. In this analysis, it quantified the sensitivity of the map in Equation (8) to noise in the observed displacements. Note that the noise considered here might stem from both image noise and errors in the estimation of the marker displacements (e.g., errors in the optical flow estimation, see Section 5).

The Monte Carlo simulations were performed as follows: (1) $N_{f}$ force samples were randomly drawn. Each of these samples represented a three-dimensional concentrated force vector, which was placed at the origin of the world coordinate frame. (2) For each force sample, $N_{c}$ marker configurations were randomly drawn for each layout class, and the resulting marker displacements were projected to the image frame. (3) Therefore, the resulting value of κ was computed and averaged for the $N_{c}$ different random marker configurations (in the same layout class).

**Figure 5:**
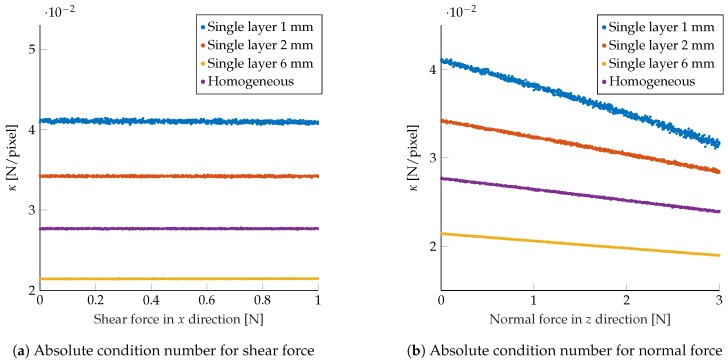
The plots show the resulting absolute condition number κ for various shear (**a**) and normal (**b**) force samples, averaged over multiple configurations in the same class, for the different layout classes.

Figure 5 shows the average magnitude of κ for different types of forces. For ease of visualization, the results presented here considered the application of pure shear (along one axis) or pure normal force, but similar conclusions were obtained for generic force vectors. The figures indicate how the map in Equation (8) was more robust for configurations with the markers placed closer to the camera, i.e., deeper in the soft material. The layout class with an homogeneous spread of markers between 1 mm and 6 mm showed higher robustness compared to the case of all markers placed at 1 mm or 2 mm depth (but was lower when compared to the class with the markers placed at 6 mm depth). Note that there were two counteracting effects in the model considered, that is, markers closer to the camera exhibited smaller displacements, but these displacements underwent higher amplification when projected to the image.

**Figure 6:**
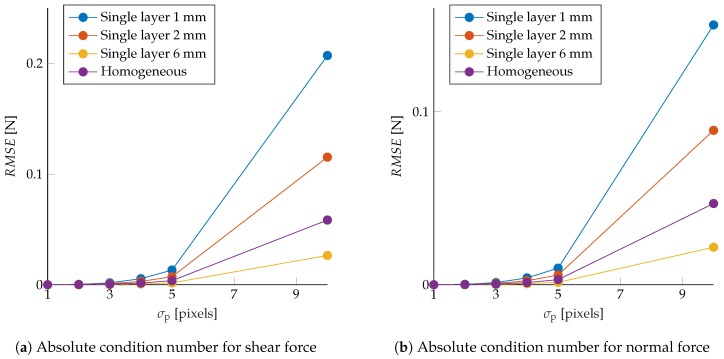
Tactile sensing applications are simulated by drawing shear (**a**) and normal (**b**) force samples, computing the resulting marker displacements, and projecting these to the image frame. Therefore, Gaussian noise with variance $\sigma_{p}^{2}$ was added to the observed displacements, and the force was reconstructed through the map in Equation (8). The resulting averaged root mean squared error is shown in these plots for different $\sigma_{p}$.

The sensor’s robustness to noise was also evaluated in the same simulation framework by perturbing the observed marker displacements after projecting these to the image frame. Zero-mean Gaussian noise with variance $\sigma_{p}^{2}$ was added to the observed Δp, and the force was reconstructed using Equation (8). The resulting root mean squared error (RMSE) for varying $\sigma_{p}$ (averaged over the different simulations) is shown in Figure 6, showing that, considering higher order terms, neglected in the linearization, led to the same trend as in Figure 5.

### 3.3. Sensor Threshold

The tactile sensor threshold is given by the minimum force that generates a noticeable change in the image, i.e., when a marker is observed at different pixel coordinates. In the worst case scenario (a similar analysis applies to other scenarios), each marker center (or feature) of interest is at the center of a pixel. In this case, a marker needs to move by at least half a pixel to be observed at a different position in the image. Therefore, during the simulations described in Section 3.2, the component-wise maximum among the observed marker displacements was computed (that is, the maximum component of the observed Δp vector). For each force sample, this quantity was averaged over the $N_{c}$ random marker configurations. The sensor threshold was then estimated as the minimum magnitude of the (shear or normal) force that yields a displacement greater than half a pixel.

Table 2 shows the resulting sensor threshold for pure normal and shear forces, respectively, for the different classes of layouts. In the simplified scenario considered, the configurations with the markers placed closer to the sensor’s top surface exhibited a smaller threshold, while the layout class with homogeneous spreads of markers at different depths retained a threshold comparable to the layout class with a single layer at 1 mm depth.

Summarizing, the analyses presented here show that spreading the markers homogeneously at different depths (in this example between 1 mm and 6 mm) might yield an advantageous trade-off between robustness to noise in the observed marker displacements and sensor threshold. In particular, compared to the case of all markers at a depth of 1 mm, this layout class showed higher robustness to noise while retaining a comparable sensor threshold (which for this example was about 2–3 times smaller than the layout with a single layer at 6 mm depth).

## 4. Ground Truth Data

In the context of supervised learning, each data point is associated with a label that represents the ground truth of the quantity of interest. In the approach presented here, a label vector representing the normal force distribution is assigned to the image that the camera captures when this force is applied. In this section, the procedure followed to collect the data, which were then processed and used to train the learning architecture presented in Section 6, is described. The generation of ground truth label vectors is then discussed.

### 4.1. Data Collection

The training data were automatically collected by pressing an indenter’s tip against the sensor gel by means of a milling and drilling machine (Fehlmann PICOMAX 56 TOP).

**Figure 7:**
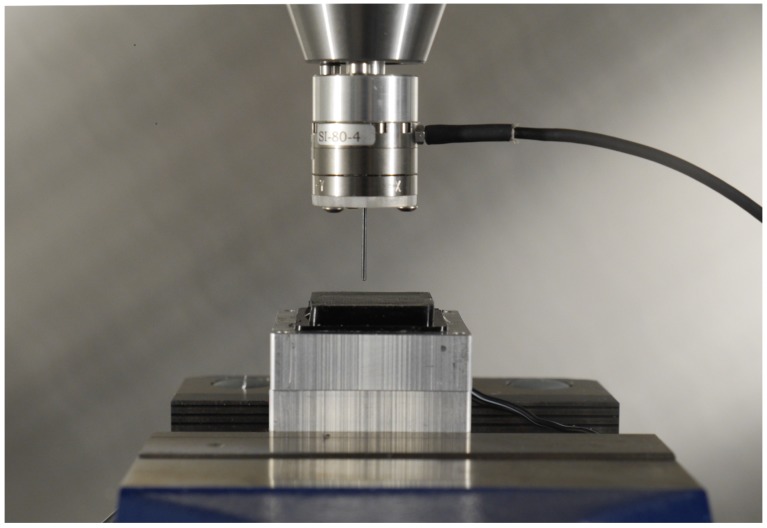
The image shows the data collection setup in the automatic milling machine. The F/T sensor is the cylindrical device connected with the cable in the upper part of the figure.

The machine was equipped with a three-axis computer numerical control, which enabled precise motion (in the order of $10^{-3}$ mm) of a spindle in the three-dimensional operating space. A spherical-ended cylindrical indenter (40 mm long, with a diameter of 1.2 mm) was mounted on a state-of-the-art six-axis F/T sensor (ATI Mini 27 Titanium) through a plexiglass plate. The F/T sensor was attached to the spindle and used to measure the force applied by the indenter, which was pressed against the gel at different depths and positions. An image of this setup is shown in Figure 7.

The milling machine outputted a digital signal, which was positive when the needle reached the commanded pressure position. The signal was read by a standard laptop, which was also responsible for reading the F/T sensor data and the camera stream. The data were synchronized on the laptop by extracting the image frames that were recorded (and cropped to 480 × 480 pixels) when the digital signal was positive. These images were then matched to the corresponding normal force value, which was sent by the F/T sensor.

### 4.2. Data Labels

**Figure 8:**
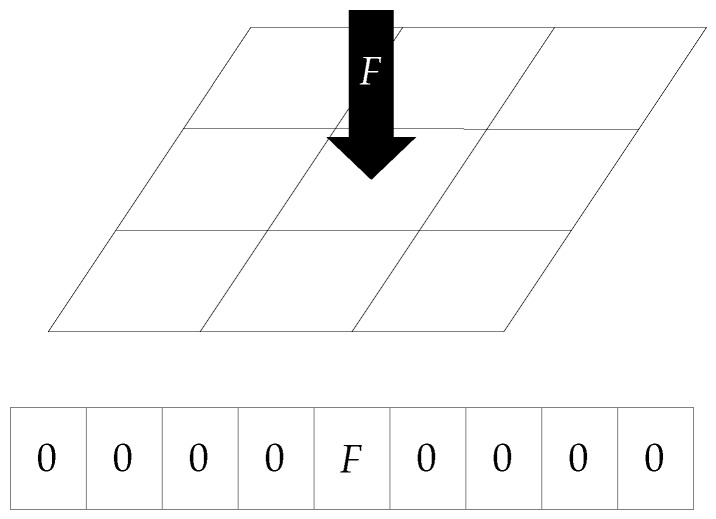
The scheme shows an example of a ground truth label vector. The indenter applies a force *F* to the center of the surface, which is part of a specific bin. The corresponding central vector component is then set to *F*, while the remaining components are set to zero.

The neural network architecture presented in Section 6 requires the labels to be expressed as real vectors. To this purpose, the sensor surface was divided into *n* bins, each of these representing a different region. Therefore, a corresponding *n*-dimensional label vector embedded the force that was applied to each of these regions. Since the force was applied to the gel with the relatively small spherical indenter used for the training data collection, this was simplified as a point force at the center of the contact. The point of application of the force was mapped to one of the *n* surface bins, and the value of the force read by the F/T sensor was assigned to the label vector component that represented the appropriate bin. All remaining components were then set to zero, to indicate a zero force distribution in those regions. A scheme of this procedure is shown in Figure 8.

Note that the number of surface bins *n* provides an indication of the spatial resolution of the sensor (measured in millimeters), which is different from the camera resolution that was fixed at 480 × 480 pixels in the scenario considered here. A larger *n* provides a finer spatial resolution, at the expense of a higher dimensional map between images and force distribution, which is generally more complex to estimate.

## 5. Optical Flow Features

Extracting meaningful features by processing the images has the benefit of reducing the required amount of training data and the training times.

In a soft material, the strain field provides information about the force applied to the surface, which generates the material deformation [25]. In the approach presented here, the spherical markers’ displacement rendered an approximation of the strain field, which could be obtained by means of optical flow techniques. In fact, the input features to the supervised learning architecture presented in Section 6 were derived from the estimated optical flow.

This section discusses two approaches to the feature engineering problem, based on sparse and dense optical flow, respectively [26].

### 5.1. Sparse Optical Flow

Sparse optical flow methods are based on tracking the movement of a set of keypoints. In particular, the Lucas Kanade algorithm (see [27]) solves the optical flow equations relying on the assumption that pixels in a small neighborhood have the same motion.

**Figure 9:**
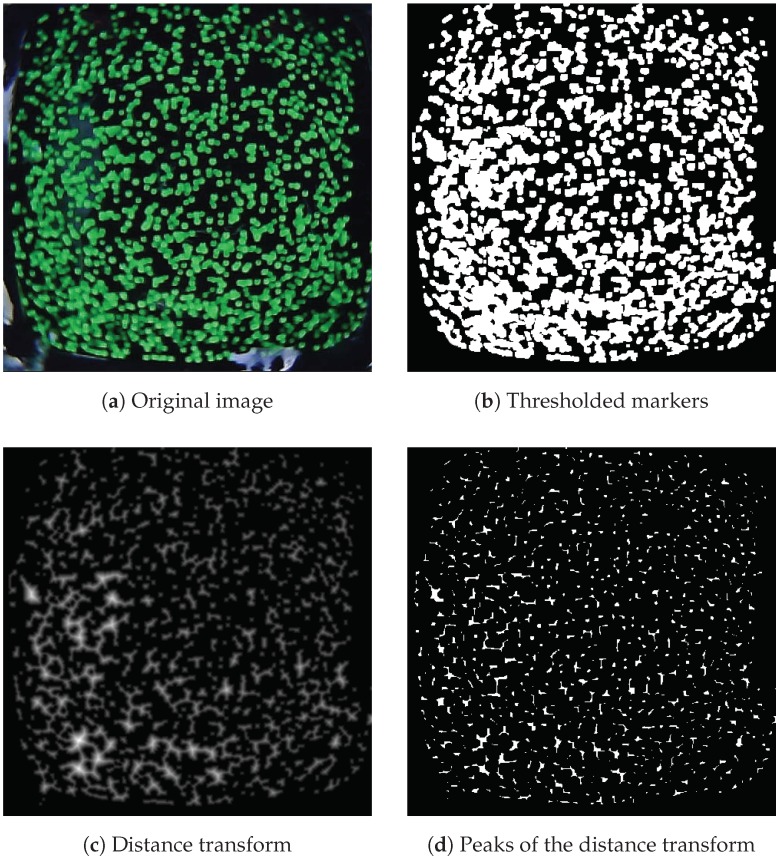

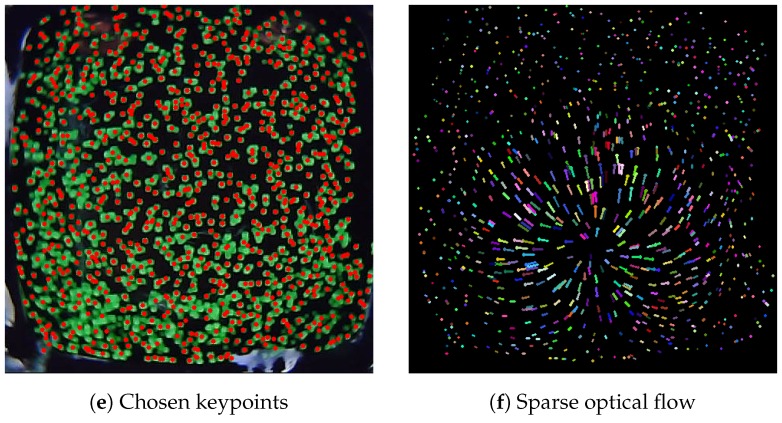
The original image (**a**) is thresholded to extract the green color of the markers (**b**). The distance transform (see [29] for the definition) is computed (**c**), and its local peaks are extracted (**d**). The watershed algorithm is finally applied to segment the original image into the different markers. The centers of the resulting contours, in red (**e**), are chosen as the keypoints to track through Lucas Kanade optical flow in the following frames (**f**).

For the tactile sensor proposed in this paper, the centers of the fluorescent markers represented a natural choice as keypoints. However, identifying these keypoints required a detection phase with the gel surface at rest. The proposed algorithm is based on the watershed transformation for image segmentation (see [28]). The different steps are explained in Figure 9.

Once the keypoints were chosen, they were tracked in the following frames using Lucas Kanade optical flow. The resulting flow was represented as a tuple of magnitude and angle with respect to a defined axis. Similar to the approach explained for generating the label vectors, the image was divided into a uniform grid of *m* regions. Note that *m* does not necessarily have to be equal to *n*. The markers were assigned to the appropriate resulting bins, depending on their location in the image. The features were then defined by the following average quantities, for each image bin i=1,⋯,m,

(11) $$\begin{array}{c} d_{\mathrm{avg},i}=\frac{1}{N_{i}}\sum_{j=0}^{N_{i}-1}d_{ij} \end{array}$$

(12) $$\begin{array}{c} \alpha_{\mathrm{avg},i}=\mathrm{atan}2\left(\sum_{j=0}^{N_{i}-1}\sin\left(\alpha_{ij}\right),\sum_{j=0}^{N_{i}-1}\cos\left(\alpha_{ij}\right)\right), \end{array}$$

where $d_{ij}$ and $\alpha_{ij}$ represent the magnitude and angle tuple relative to the displacement of the marker *j* in the region *i*, and $N_{i}$ is the number of markers assigned to the region *i*. The average defined in Equation (12) is often referred as the circular mean of the angles (see [30] (p. 106) for an explanation).

Under the assumptions that the markers were homogeneously distributed over the entire volume of the gel, these average quantities provided an approximation of the optical flow at each of the bin centers. The feature vector for each image was therefore obtained by appropriately stacking the two average quantities for each of the *m* regions, resulting in a 2m-dimensional feature space. Note that the averages also provided invariance of the features to the marker pattern, provided that the same material and design were retained.

### 5.2. Dense Optical Flow

Compared to sparse optical flow, dense optical flow methods are more accurate, at the expense of a higher need for computational resources [31]. Moreover, they do not rely on a preliminary detection phase, which may introduce further inaccuracies (see, for example, the lower-left part of Figure 9e). In fact, rather than tracking a set of features over subsequent frames, they aim at estimating the motion at each pixel of the image. The Dense Inverse Search (DIS) optical flow algorithm (see [32]) reconstructs the motion through the computation of the flow at different image scales, and exploits an inverse search technique to obtain a considerable increase in speed. Therefore, rather than relying on the detection of keypoints, the DIS algorithm reconstructs the optical flow from any trackable distinct pattern. Moreover, it exploits the full resolution of the camera (distinct from the spatial resolution of the tactile sensor), rendering motion information at each pixel of the image. As a consequence, the spatial resolution of the tactile sensor is not limited by the number of markers. Note that a specific marker layout (e.g., a uniform grid) would not simplify the image processing and the reconstruction of the dense optical flow, and an eventual regular spacing could cause the loss of information at certain pixels. Conversely, the random spread of markers facilitates manufacture and does not impact the deployment of the sensor to applications where a larger contact surface with arbitrary shape is required.

**Figure 10:**
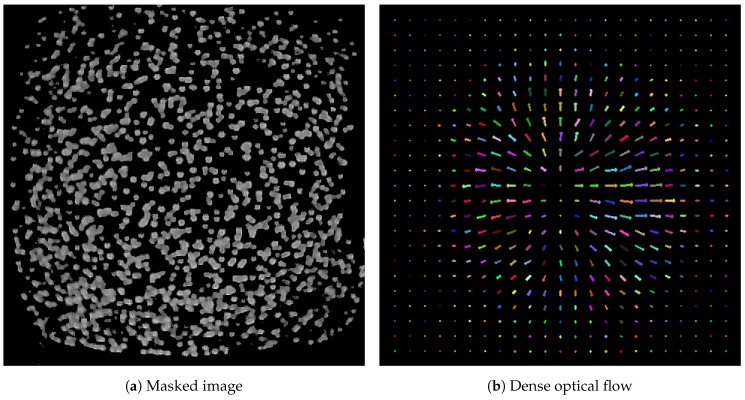
The original image is processed by replacing non-green regions with black pixels (**a**). The DIS algorithm computes the dense optical flow (**b**) on the resulting image. Note that the flow is estimated at each pixel, and a subsampled version is shown in (**b**) for ease of visualization.

To match the required format of the DIS algorithm, the original image was processed. The green regions in the image were thresholded and the remaining non-green regions were replaced with black pixels, removing external light disturbances and material imperfections. Finally, the resulting image was converted to gray-scale. An example of the masked image and the computed dense optical flow are shown in Figure 10.

As in the case of the sparse keypoint tracking, the resulting flow was represented as tuples of magnitude and angle, in this case for each pixel. The pixels were then assigned to *m* image bins. Redefining $N_{i}$ for the dense optical flow case as the number of pixels assigned to the region *i*, the averages defined in Equations (11) and (12) were computed, to compose a set of 2m features.

## 6. Learning Architecture

**Figure 11:**
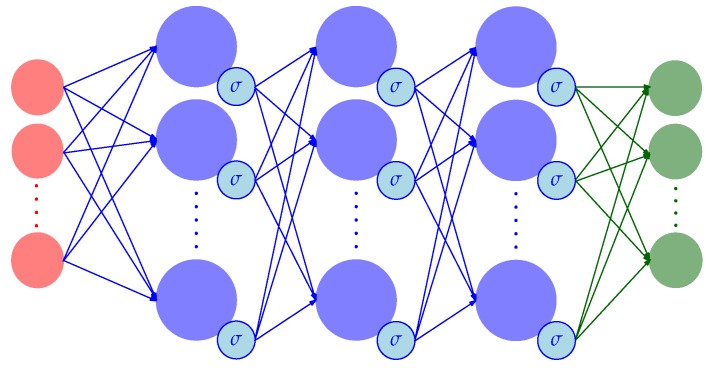
The figure shows a schematic representation of the DNN architecture, neglecting the bias terms. The 2m input layer neurons (in red) enter the hidden layers (in blue), which have sigmoid activation functions. The output layer, with *n* outputs, is shown in green. Note that 2m and *n* are the number of features and surface bins, respectively.

The problem of reconstructing the normal force distribution from images can be formulated as a multiple multivariate regression problem, that is, a regression problem where both input features and labels are multi-dimensional vectors. A multi-output deep neural network (DNN) provides a single function approximation to the underlying map, which exploits the interrelations among the different inputs and outputs. Similar to the work in [6], the approach proposed in this article presents a feedforward DNN architecture (see Figure 11), with three fully connected hidden layers of width 1600, which apply a sigmoid function to their outputs. The input data to the network were the 2m (sparse or dense) optical flow features described in Section 5, while the output vectors provided an estimate of the normal force applied at each of the *n* bins the tactile sensor surface was divided into, as described in Section 4.

The architecture weights were trained with RMSProp with Nesterov momentum (known in the literature as Nadam, see [33]), with training batches of 100 samples and a learning rate of 0.001. The solver aimed at minimizing an average root mean squared error (aRMSE), defined according to Borchani et al. [34] as,

(13) $$\begin{array}{c} aRMSE=\frac{1}{n}\sum_{i=0}^{n-1}\sqrt{\frac{\sum_{l=0}^{N_{\mathrm{set}}-1}{\left(y_{i}^{\left(l\right)}-{\hat{y}}_{i}^{\left(l\right)}\right)}^{2}}{N_{\mathrm{set}}}}, \end{array}$$

where $y_{i}^{\left(l\right)}$ and ${\hat{y}}_{i}^{\left(l\right)}$ denote the *i*th true and predicted label vector component, respectively, for the *l*th sample in the considered dataset portion (i.e., training, validation, and test sets), which contains $N_{\mathrm{set}}$ data points.

Twenty percent of each dataset was retained as a test set, against which the performance was evaluated. Ten percent of the remaining data points were randomly chosen as a validation set. Dropout regularization (at a 10% rate) was employed at each hidden layer during training, which terminated when the loss computed on the validation set did not decrease for 50 consecutive epochs.

Due to the very sparse structure of the label vectors considered in the experiments presented here (see Figure 8), two evaluation metrics were computed on the test set, in addition to the aRMSE, which are more intuitive for this application. For $l=0,1,\cdots ,N_{\mathrm{set}}-1$, the label component of the ground truth vector where the magnitude of the applied force is maximum was computed,

(14) $$\begin{array}{c} k_{l}=\underset{i=0,1,\cdots ,n-1}{arg max}\left|y_{i}^{\left(l\right)}\right|. \end{array}$$

The same quantity was computed for the estimated label vector,

(15) $$\begin{array}{c} {\hat{k}}_{l}=\underset{i=0,1,\cdots ,n-1}{arg max}\left|{\hat{y}}_{i}^{\left(l\right)}\right|. \end{array}$$

Denoting c(k) as the location of the center of a surface bin *k* on the horizontal plane, a distance metric was introduced,

(16) $$\begin{array}{c} d_{\mathrm{loc}}=\frac{1}{N_{\mathrm{set}}}\sum_{l=0}^{N_{\mathrm{set}}-1}{∥c\left(k_{l}\right)-c\left({\hat{k}}_{l}\right)∥}_{2}. \end{array}$$

Finally, an error computed on the maximum components of the true and estimated label vectors was defined as,

(17) $$\begin{array}{c} RMSE_{\mathrm{mc}}=\sqrt{\frac{1}{N_{\mathrm{set}}}\sum_{l=0}^{N_{\mathrm{set}}-1}{\left(y_{k_{l}}^{\left(l\right)}-{\hat{y}}_{{\hat{k}}_{l}}^{\left(l\right)}\right)}^{2}}. \end{array}$$

The metric in Equation (16) provides an indication of how close the location of the maximum estimated force was to the real location of the force applied by the indenter described in Section 4. The metric in Equation (17) indicates how accurate (in the magnitude) the estimation of this force was.

## 7. Results

The tactile sensor’s performance was evaluated on a dataset collected on a test indenter, as described in Section 4. The images (acquired at a resolution of 640 × 480 pixels) were cropped to a region of interest of 480 × 480 pixels, which covered the gel surface. A total of 10,952 data points were recorded by commanding the needle to reach various depths (up to 2 mm, for a maximum force of 1 N) at different positions on the surface, which were defined by an equally-spaced grid (0.75 mm between adjacent points). Note that other ranges of force could be similarly covered by replacing the Ecoflex™ GEL with another material with different hardness.

**Figure 12:**
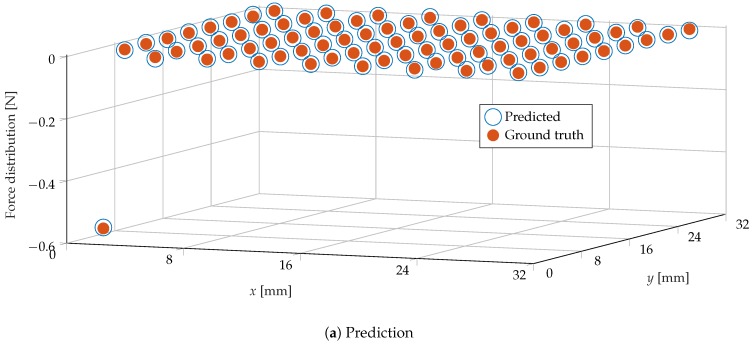

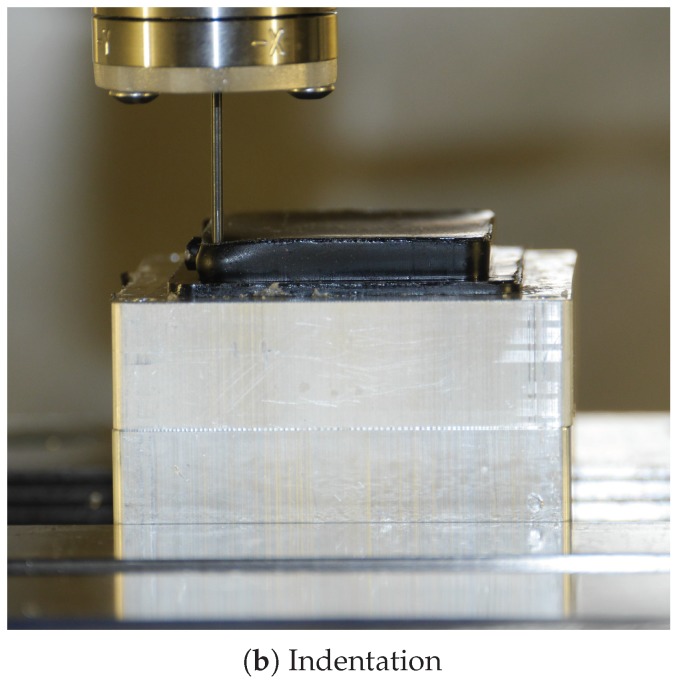
(**a**) The prediction of the force distribution for a sample in the test set (for a number of image bins *m* = 1600, surface bins *n* = 81 and dense optical flow features); and (**b**) the corresponding indentation. High accuracy was achieved at each bin, including the corners of the gel, which underwent a deformation that generally differed from the rest of the surface when subject to force (due to boundary effects).

**Figure 13:**
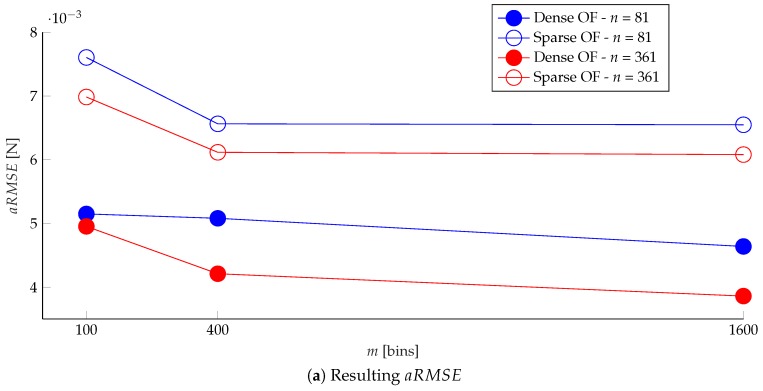

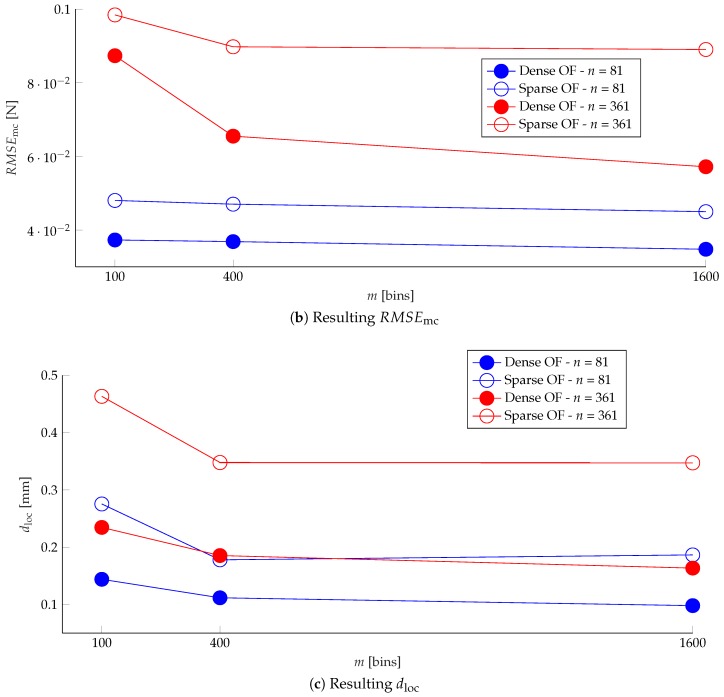
The plots show the resulting metrics (defined in Equations (13), (17) and (16)) for various values of image bins *m* and surface bins *n*, and for dense and sparse optical flow (OF) features. The resolution of the ground truth F/T sensor is 0.06 N.

An example of the prediction of the normal force distribution is shown in Figure 12. The learning architecture was evaluated for different values of *m* and *n*, and for features based on sparse and dense optical flow. The results, greatly outperforming the authors’ previous work in [6], are shown in Figure 13.

The plots show that, in terms of the aRMSE, which is the loss actually optimized during training, the dense optical flow outperformed the sparse optical flow in estimating the force distribution, in both the finer and coarser spatial resolution cases (determined by *n*). However, this metric did not provide a reliable comparison across different spatial resolutions, since it was influenced by the zero values in the label vectors, which were considerably more in the finer resolution case.

As shown in Figure 13b, the $RMSE_{\mathrm{mc}}$ decreased by increasing the number of averaging regions in the image, and was lower for the dense optical flow case, considering the same number of surface bins. Estimating the force distribution with a finer spatial resolution slightly degraded the performance according to this metric, and might require a different network architecture and more training data, due to the higher dimension of the predicted output.

The use of the information at all pixels, provided by the dense optical flow, resulted in a better accuracy in estimating the location of the applied force (indicated by $d_{\mathrm{loc}}$), compared to the sparse optical flow. In fact, the performance obtained with finer resolution and dense optical flow features was comparable to the case with coarser resolution and sparse optical flow features. Moreover, excessively increasing the number of image bins *m* might degrade the performance for the sparse optical flow case, since some averaging regions might not be covered by a sufficient number of detected keypoints.

## 8. Conclusions

This article has discussed the design of and the motivation for a novel vision-based tactile sensor, which uses a camera to extract information on the force distribution applied to a soft material. Simulation results have shown the benefits of the proposed strategy. The experimental results presented in this paper (see Supplementary video) have shown high accuracy in reconstructing the normal force distribution applied by a small spherical indenter. Although in the experimental setup considered an approximation to a point indenter has been applied, the proposed learning algorithm does not explicitly use knowledge of this information (that is, the point force and the zero valued regions are indistinctly predicted through the same architecture). In fact, the representation of the force distribution introduced in this article (embedded in appropriate label vectors) is suitable to represent generic normal forces, including multi-point contacts and larger indentations. Moreover, this representation can be readily extended to shear forces, by appropriate vector concatenation. Future work will investigate the cases not considered in this article, to address the limitations introduced by the point force approximation. To this purpose, the approach proposed here will need to be evaluated for arbitrary force distributions on a wider dataset, with various indenters and multiple types of contact. These steps will be aimed towards the generalization of the current approach to touch with objects of arbitrary shapes (e.g., for robotic grasping tasks).

Dense optical flow features, whose application in the context of vision-based tactile sensors is novel (to the authors’ knowledge), exhibit higher performance compared to (sparse) keypoint tracking and retain fast execution times. The use of these features leverages the randomized marker layout, since (as highlighted in Section 5) the spacing required for regular grid patterns, as for example in [35], might instead cause a decrease in accuracy in the estimation of the dense optical flow.

The spatial resolution (n=361 corresponds to surface bins with a side of 1.58 mm) and sensing range achieved are comparable to the human skin (see [36] for a reference), and the resulting pipeline runs in real-time at 60 Hz.

## Figures and Tables

**Table 1:** Simulation parameters.

Symbol	Value	Description
ν	0.4999	Poisson’s ratio
*E*	0.1 MPa	Young’s modulus
*f*	256 pixels	Focal length
*d*	20 mm	*z*-coordinate of the camera optical center
$N_{f}$	1000	Number of drawn force samples
$N_{c}$	1000	Number of drawn configurations for each class

**Table 2:** Sensor threshold for various layout classes.

Layout Class	Sensor Threshold (Shear)	Sensor Threshold (Normal)
Single layer at 1 mm	0.013 N	0.035 N
Single layer at 2 mm	0.027 N	0.059 N
Single layer at 6 mm	0.056 N	0.084 N
Homogeneous spread	0.018 N	0.048 N

## Supplementary Materials

The following are available online at https://www.mdpi.com/1424-8220/19/4/928/s1, Video S1: Design, motivation and evaluation of a full-resolution optical tactile sensor.

## Appendix A. Choice of the Particles’ Density

The cure time of the Ecoflex™ GEL, which is mixed with the markers, is about 2 h at a room temperature of 23 ${}^{{}^{\circ}}$C. However, after pouring the material into the mold, the cure is accelerated with mild heat (up to 80 ${}^{{}^{\circ}}$C in an oven), resulting in cure times of about 10 min. During this time, a density difference between the spherical particles and the material might lead to sinking or floating of the markers.

Assuming that a particle has higher density than the fluid (a symmetrical analysis applies to the opposite case), the dynamics of a single marker in the material is described by the following differential equation,

(A1) $$\begin{array}{c} \rho_{p}V_{p}{\ddot{x}}_{p}\left(t\right)=\rho_{p}V_{p}g-\rho_{f}V_{p}g-F_{D}\left(t\right), \end{array}$$

where t≥0 denotes the time in seconds; $x_{p}$ indicates the position of the particle (positive in the direction of gravity); $\rho_{p}$ and $\rho_{f}$ denote the density of the particle and the fluid, respectively; $V_{p}$ is the volume of the particle; and *g* is the gravitational acceleration. Given the low Reynolds number of the considered application (low speed and very small particle diameter), the drag force $F_{D}$ is described through the Stoke’s law, that is,

(A2) $$\begin{array}{c} F_{D}\left(t\right)=6\pi \eta R_{p}{\dot{x}}_{p}\left(t\right), \end{array}$$

where η is the dynamic viscosity of the material and $R_{p}$ is the radius of the particle.

The solution to the differential equation in Equation (A1) is,

(A3) $$\begin{array}{c} x_{p}\left(t\right)=\left(\rho_{p}-\rho_{f}\right)V_{p}g\left[t+\frac{\rho_{p}V_{p}}{b}\left(\exp\left(-\frac{bt}{\rho_{p}V_{p}}\right)-1\right)\right]+x_{p,0}, \end{array}$$

with $b:=6\pi \eta R_{p}$, and $x_{p,0}$ the starting position of the marker. The parameters of the setup presented in this article are shown in Table A1, and lead to a change in position of about 0.35 mm in 10 min. Considering that the mold lays on one of its sides during the material cure, this change does not affect the marker distribution to a large extent. Moreover, for simplicity, the drastic increase in dynamic viscosity (until hardening) that the material experiences during the cure was not considered in this analysis (which considers η constant). In practice, the dynamic viscosity increase contributes to considerably slow down the particle, further reducing the resulting displacement to a negligible value.

**Table A1:** Physical parameters.

Symbol	Value	Description
$\rho_{p}$	1020 Kg/m${}^{3}$	Density of a marker
$\rho_{f}$	980 Kg/m${}^{3}$	Density of the material
$V_{p}$	0.0654 mm${}^{3}$	Volume of a marker
*g*	9.81 m/s${}^{2}$	Gravitational acceleration
η	9.3 Pa·s	Dynamic viscosity of the material
$R_{p}$	0.25 mm	Radius of a marker

## Appendix B. Model Derivation

From Equations (4) and (5), the difference between the camera matrices after and before displacement is,

(A4) $$\begin{array}{c} K_{a,j}-K_{b,j}=\left[\begin{array}{ccc} \frac{fH_{j,3}F}{\left(d-z_{j}-H_{j,3}F\right)\left(d-z_{j}\right)} & 0 & 0\\ 0 & \frac{fH_{j,3}F}{\left(d-z_{j}-H_{j,3}F\right)\left(d-z_{j}\right)} & 0 \end{array}\right]. \end{array}$$

Therefore, Equation (3) becomes,

(A5) $$\begin{array}{cc} \Delta p_{j} & =\frac{fH_{j,3}F}{\left(d-z_{j}-H_{j,3}F\right)\left(d-z_{j}\right)}\left[\begin{array}{c} x_{j}\\ y_{j} \end{array}\right]+\frac{f}{d-z_{j}-H_{j,3}F}\left[\begin{array}{c} H_{j,1}F\\ H_{j,2}F \end{array}\right]\\ & =\frac{1}{\left(d-z_{j}-H_{j,3}F\right)\left(d-z_{j}\right)}\left\{fH_{j,3}F\left[\begin{array}{c} x_{j}\\ y_{j} \end{array}\right]+f\left(d-z_{j}\right)\left[\begin{array}{c} H_{j,1}F\\ H_{j,2}F \end{array}\right]\right\}. \end{array}$$

Assuming that a marker is never at the same location as the optical center, Equation (A5) can be rearranged as,

(A6) $$\begin{array}{cc} \left(d-z_{j}-H_{j,3}F\right)\left(d-z_{j}\right)\Delta p_{j} & =fH_{j,3}F\left[\begin{array}{c} x_{j}\\ y_{j} \end{array}\right]+f\left(d-z_{j}\right)\left[\begin{array}{c} H_{j,1}F\\ H_{j,2}F \end{array}\right] \end{array}$$

(A7) $$\begin{array}{cc} \left[{\left(d-z_{j}\right)}^{2}-H_{j,3}F\left(d-z_{j}\right)\right]\Delta p_{j} & =fH_{j,3}F\left[\begin{array}{c} x_{j}\\ y_{j} \end{array}\right]+f\left(d-z_{j}\right)\left[\begin{array}{c} H_{j,1}F\\ H_{j,2}F \end{array}\right] \end{array}$$

(A8) $$\begin{array}{cc} {\left(d-z_{j}\right)}^{2}\Delta p_{j} & =H_{j,3}F\left(d-z_{j}\right)\Delta p_{j}+fH_{j,3}F\left[\begin{array}{c} x_{j}\\ y_{j} \end{array}\right]+f\left(d-z_{j}\right)\left[\begin{array}{c} H_{j,1}F\\ H_{j,2}F \end{array}\right] \end{array}$$

(A9) $$\begin{array}{cc} \Delta p_{j} & =\left\{\frac{\Delta p_{j}}{d-z_{j}}H_{j,3}+\frac{f}{{\left(d-z_{j}\right)}^{2}}\left[\begin{array}{c} x_{j}\\ y_{j} \end{array}\right]H_{j,3}+\frac{f}{d-z_{j}}\left[\begin{array}{c} H_{j,1}\\ H_{j,2} \end{array}\right]\right\}F, \end{array}$$

which is the same expression as in Equation (6).
